# Identification of Optimal Reference Genes for Expression Analysis in Radish (*Raphanus sativus* L.) and Its Relatives Based on Expression Stability

**DOI:** 10.3389/fpls.2017.01605

**Published:** 2017-09-15

**Authors:** Mengmeng Duan, Jinglei Wang, Xiaohui Zhang, Haohui Yang, Haiping Wang, Yang Qiu, Jiangping Song, Yangdong Guo, Xixiang Li

**Affiliations:** ^1^Key Laboratory of Biology and Genetic Improvement of Horticultural Crops, Ministry of Agriculture, Institute of Vegetables and Flowers, Chinese Academy of Agricultural Sciences Beijing, China; ^2^Department of Vegetable Sciences, College of Horticulture, China Agricultural University Beijing, China

**Keywords:** radish, Chinese cabbage, distant hybrid, organs, stress, pistil development, RT-qPCR, reference gene

## Abstract

Radish (*Raphanus sativus*) is an important cruciferous root crop with a close relationship to Chinese cabbage (*Brassica rapa*). RT-qPCR is used extensively to evaluate the expression levels of target genes, and accurate measurement of target gene expression with this method is determined by the valid reference genes used for data nomalization in different experimental conditions. Screening for appropriate reference genes with stable expression based on RT-qPCR data is important for gene expression and functional analysis research in radish and its relatives. However, many researches have thought that almost no single reference gene is widely suitable for all experimental conditions, and few researchers have paid attention to the validation of reference genes in radish gene expression analysis. In the present study, 12 candidate reference genes were selected for analysis. Their expression in 28 samples, including 20 radish samples from different organs and conditions, four Chinese cabbage organs and four organs of their distant hybrid, was assessed by RT-qPCR and then five software tools—ΔCt, geNorm, NormFinder, BestKeeper and RefFinder—were used to compare their expression stability. The results showed that the most suitable reference genes were different in different organs and conditions. *GAPDH, DSS1*, and *UP2* were optimal reference genes for gene expression analysis in all organs and conditions in radish. *UPR, GSNOR1*, and *ACTIN2/7* were the most stable reference genes in different radish organs. *UP2* and *GAPDH* were suitable reference genes for radish pistil development studies. *RPII, UBC9*, and *GAPDH* had the most stable expression in radish under various stresses. *DSS1, UP2*, and *TEF*_*2*_ were the optimal reference genes for Chinese cabbage organs, whereas *TUA* was optimal for the distant hybrid. *UP2*, and *TEF*_*2*_ were appropriate reference genes for all of the samples together. The optimal reference genes we identified, *UP2, GAPDH, UPR*, and *GSNOR1* were verified by normalizing the expression patterns of *YAB3, RPL*, and *FUL*. These results will provide important information for selecting target reference genes in different research contexts and improve the accuracy and precision of gene expression analysis for radish, Chinese cabbage and their distant hybrid.

## Introduction

RT-qPCR (Quantitative Real-time PCR) is an important and effective method to evaluate the expression of target genes in different tissues, organs and conditions (Bustin, 2000, 2002; Gachon et al., 2004). Although RT-qPCR has many advantages including rapidity, sensitivity, and specificity, the expression data are affected by experimental conditions or inherent technical variations as well as true biological variation (Bustin, 2002; Derveaux et al., 2010). Stably expressed reference genes are usually used to normalize transcriptome quantification through exposure to the same preparation processes as the target genes. Therefore, appropriate reference genes for data normalization are critical to obtain accurate expression data by RT-qPCR (Ginzinger, 2002; Gachon et al., 2004; Dheda et al., 2005; Guenin et al., 2009; Schmidt and Delaney, 2010). Otherwise, inappropriate reference genes will lead to errors in the expression data for the target gene (Dheda et al., 2005). An appropriate reference gene should be expressed stably in different types of cells, tissues and organs, and at different developmental stages. At the same time, its expression should be high or moderate and assumed to be unaffected under different experimental conditions (Bustin, 2002; Brunner et al., 2004; Czechowski et al., 2005; Gutierrez et al., 2008).

The most commonly used reference genes for the normalization of RT-qPCR data in plants include glyceraldehyde-3-phosphate dehydrogenase (*GAPDH*), actin (*ACT*), ubiquitin (*UBI*), 18S ribosomal RNA (*18S*), α-tubulin and β-tubulin (*TUA* and *TUB*, respectively), and the ubiquitin-conjugating enzyme (*UBC*), which function in maintaining cell survival irrespective of physiological conditions (Bustin, 2002; Brunner et al., 2004; Radonić et al., 2004; Czechowski et al., 2005; Jain et al., 2006; Gutierrez et al., 2008). These reference genes were identified by Northern-blotting in the pre-genomic era and were assumed to have stable expression at various developmental stages and under various experimental conditions (Jain et al., 2006). Unfortunately, several studies have proved that the transcript levels of these traditional reference genes vary under different experimental conditions (Thellin et al., 1999; Suzuki et al., 2000; Czechowski et al., 2005; Zhu et al., 2011). Thus, many validation methods have been used to confirm the variability of conventional reference genes though systematic research in various plants, such as *Arabidopsis thaliana* (Han et al., 2013), potato (Castro-Quezada et al., 2013), tomato (Expósitorodríguez et al., 2008), cabbage (Chen et al., 2010), soybean (Bo et al., 2008), tobacco (Schmidt and Delaney, 2010), watermelon (Kong et al., 2014), melon (Kong et al., 2016), pearl millet (Shivhare and Lata, 2016), and celery (Li et al., 2016b). These experiments show that no gene has universal expression in all tissues types or under all experiment conditions. Thus, one or more different reference genes need to be selected according to the specific set of biological samples being studied.

With the development of molecular biological technologies such as the Affymetrix GeneChip, microarrays and high-throughput sequencing technologies in recent years, a large number of novel reference genes have been developed in *A. thaliana* (Czechowski et al., 2005) and crops such as *Brassica juncea* (Qi et al., 2010), *Brassica napus* (Yang et al., 2014), papaya (Zhu et al., 2011) and rice (Jain, 2009). In one study, an F-box protein (F-box), a *SAND* family protein and the mitosis protein *YLS8* were expressed more stably than the traditional reference genes *ACTIN-2*, elongation-factor-1-a (*EF1-*α) and ubiquitin-conjugating enzyme 10 (*UBC10*) in *A. thaliana* (Remans et al., 2008). *UBC9* and *UP2* were chosen as reference genes on the basis of microarray data described by Schmid et al. in tobacco, and have also shown more stable expression than some traditional reference genes in other crops (Czechowski et al., 2005; Schmid et al., 2005; Kwon et al., 2009; Kumar et al., 2011). Twelve novel reference genes obtained from genomic and transcriptomic data were identified to perform better than the traditional reference genes *ACTIN7* and *GAPDH* in *B. napus* (Yang et al., 2014). Additionally, the most commonly used tools to assess the stability of reference genes in a set of samples are the delta cycle threshold (ΔCt) method (Silver et al., 2006) and the software tools geNorm (Vandesompele et al., 2002), NormFinder (Andersen et al., 2004), BestKeeper (Pfaffl et al., 2004), and RefFinder (Xie et al., 2012). The integrated application of these software tools has improved the accuracy of candidate reference gene identification (Hao et al., 2014; Niu L. et al., 2015).

Radish (*Raphanus sativus* L.), a member of the Cruciferae family, is a widely cultivated root vegetable across the world. It is also a donor of elite genes for the genetic improvement of other cruciferous crops. Many researchers have worked on the selection of appropriate reference genes in Cruciferae plants, such as *Brassica rapa* in different tissues and under different abiotic and biotic stresses (Xiao et al., 2012; Wang et al., 2016), *B. juncea* in different developmental stages and under hormone treatments and drought stress (Chandna et al., 2012), and *B. napus* in different tissues (Chen et al., 2010). Few researchers have paid attention to the validity of reference genes in radish gene expression analysis, although many studies on gene expression in radish have been carried out in recent years. At present, the most commonly used reference gene in radish is still the traditional gene *ACTIN2/7* (Xu et al., 2013; Wang et al., 2014; Yu et al., 2015) though microarray and next-generation sequencing (NGS) have been applied in radish. Therefore, it is urgent to identify optimal reference genes for RT-qPCR normalization in radish, and even its distant hybrids (such as RRAA, *n* = 38, a hybrid of Chinese cabbage (*B. rapa*; AA, *n* = 10) and radish (RR, *n* = 9), which are important materials for research related to genome evolution, genetic variability, gene exchange and germplasm enhancement in Cruciferae (Lange et al., 1989; Peterka et al., 2004; Lee et al., 2011).

The silique is an important organ for oilseed production and the main reproductive organ in radish. Some research in *Arabidopsis* suggests that MADS-box genes play an important role in pistil development and molecular interactions (Alvarezbuylla et al., 2010). The establishment of floral organ polarity leads to the expression of *YABBY3* (*YAB3*) on one side of an organ, and *YAB3* influences the formation of valve margin tissue. *FRUITFUL* (*FUL*) encodes a MADS-domain transcription factor that is expressed in the valves, and has multiple functions during cell differentiation, promoting cell expansion and inhibiting cell division in specific cell types (Gu et al., 1998), and *REPLUMLESS* (*RPL*) encodes a BELL-family homeodomain transcription factor. The expression of the valve margin identity genes is limited to the valve margin through negative regulation by *FUL* in the valves and *RPL* in the replum (Roeder et al., 2003).

In order to determine suitable reference genes, or a combination of the most stable reference genes for accurate quantification of target genes in radish and its relatives, we selected traditional candidate reference genes from previous studies and the new ones from our preliminary analysis of gene stability based on different sets of transcriptome data for further analysis in the present research. The traditional nine genes widely used in radish and other crops included *GAPDH* (glyceraldehyde-3-phosphate-dehydrogenase), *TEF*_*2*_ (translation elongation factor 2), *ACTIN2/7, TUA* (tubulin alpha-5), *TUB* (tubulin beta-1), *RPII* (RNA polymerase-II transcription factor), *18S rRNA* (*18S* ribosomal RNA), *UBC9* (ubiquitin-conjugating enzyme 9) and *UP2* (uncharacterized conserved protein), and three new genes screened out by radish transcriptome analysis were *GSNOR* (GroES-like zinc-binding dehydrogenase family protein), *UPR* (uncharacterized protein family), and *DSS1* (deletion of SUV3 suppressor 1(I)). RT-qPCR was used to validate the applicability of these genes in different tissues and organs, at different development stages, and under different stress conditions in radish, Chinese cabbage and their distant hybrid. Furthermore, the expression patterns of three key genes, *YAB3, RPL*, and *FUL*, which are thought to be related to silique development in *Arabidopsis* and *Brassica* plants, were analyzed as a case study to investigate the efficiency of the reference genes. These results will be helpful for the selection of target reference genes to ensure accuracy and precision in gene expression analysis in different research contexts for radish, Chinese cabbage and their distant hybrids.

## Materials and methods

### Plant materials and treatments

The whole genome-sequenced radish inbred line “36-2,” the Chinese cabbage inbred line “chiifu” and their distant hybrid (RRAA) were used as study materials. Geminating seeds were vernalized at 4°C for about 30 days, sown in plastic pots containing a soil/vermiculite mixture (3:1) and grown in a greenhouse at the research station of the Institute of Vegetables and Flowers, Chinese Academy of Agricultural Sciences, Beijing, China. Radish seedlings with two true leaves were exposed to the following biotic and abiotic treatments. For TuMV infection treatment, a virus inoculum was prepared by homogenizing infected fresh leaves in a homogenizer with four volumes (w/v) of 0.05 M phosphate buffer (pH 7.0). The inoculation of TuMV into radish was performed as described by Zhang et al. (2007). Under a 12 h diurnal light cycle, the temperature was maintained at 25–28°C in the daytime and 20–22°C at night in an incubator. Leaf samples were obtained on 5th, 10th, and 15th days after inoculation. For pest treatment, diamondback moths were maintained on cabbage (*Brassica oleracea*) plants in a climate-controlled room at 25°C with a 12:12 h photoperiod and 50–60% relative humidity, and four diamondback moths (second or third instars) were placed on each leaf of the radish seedlings. Leaves exposed to diamondback moths were harvested at 0, 4, 24, and 48 h after infestation (Wei et al., 2013). For cold treatment, radish seedlings were stored at 2–4°C in an incubator under a 12 h diurnal light cycle for 24 h and leaf samples were collected on the 2nd, 4th, and 6th days after exposure to low temperature. For the biotic and abiotic stress treatments, leaf samples collected from two-true-leaf seedlings under normal conditions were used as a control. To examine expression during radish pistil development, flower buds of 5 mm in length and siliques at the 0th, 5th, 15th, and 30th days after pollination were collected at different radish reproductive stages under normal growth conditions. To examine expression in different organs, root, stem and leaf samples of radish plants were collected at the vegetative phase and calyx, petal, stamen and pistil samples of radish, Chinese cabbage and their distant hybrid were collected during the flowering period.

All 28 samples had three biological replicates and each replicate was obtained from three plants. The samples were immediately frozen in liquid nitrogen and stored at −80°C for further use.

### Candidate reference gene selection and primer design

Seven traditional candidate reference genes (*GAPDH, RPII, ACTIN2/7, TEF*_2_, *18S, TUA*, and *TUB*) were selected from previous studies on radish (Xu et al., 2012) and other crops (Jain et al., 2006; Remans et al., 2008; Castro-Quezada et al., 2013; Han et al., 2013). The reference genes *UBC9* and *UP2*, which have been used in some plants (Czechowski et al., 2005; Schmid et al., 2005; Kwon et al., 2009; Kumar et al., 2011) but not in radish, were also selected.

For the selection of new candidate genes, we analyzed publically transcriptomic data from the following 21 different radish tissues and organs at different developmental stages: 7, 14, 20 days root and leaf, 40, 60, 90 days cortical, cambium, xylem, root tip and leaf (Mitsui et al., 2015; http://www.nodai-genome-d.org/download.html). A candidate reference gene was defined as a gene with the most constant expression level, i.e., a gene with a small coefficients of variation (CVs) (De Jonge et al., 2007). Therefore, the raw RNA-seq data were used to calculate the mean expression value, standard deviation, and CVs according to the following formula firstly: CVs = standard deviation of RPKM/average of RPKM. Besides the stability of gene expression level, the expression intensity of candidate reference genes is also significant. Genes with high or lowly expression abundance are not appropriate for being the reference genes (Xu et al., 2015). Hence, these selected new genes had to meet the following requirements: CV ≤ 30%, 100 ≤ RPKM ≤ 500.

The coding DNA sequences (CDS) and DNA sequences of *GSNOR1, UPR, DSS1, UBC9*, and *UP2* were obtained from previously reported radish genomic data (Mitsui et al., 2015) by homology analysis. Primers for these genes were designed using Primer 5.0 for RT-qPCR. The product sizes were set in the range of 80–200 bp. At least one primer in each pair spanned the exon-intron junction to avoid amplification of gDNA in possibly contaminated samples. A single band of expected size in 1% agarose gel electrophoresis and a single peak in RT-qPCR melting curve were used as criteria to ensure the specificity of amplification for every candidate reference gene. In addition, primers for the seven traditional reference genes (*GAPDH, RPII, ACTIN2/7, TEF*_2_, *18S, TUA*, and *TUB*) were used as previously reported (Xu et al., 2012).

### Total RNA extraction and cDNA synthesis

Total RNA was extracted from all samples using an RNAprep pure Plant Kit (TransGen, Beijing, China) according to the manufacturer's instructions. Genomic DNA was eliminated from the total RNA using RNase-free DNase I. The integrity of the RNA was checked by electrophoresis in a 2% agarose gel. The quantity and purity of the RNA were evaluated using a NanoDrop™ 2000 spectrophotometer (Thermo Scientific). Samples with A_260_/A_280_ > 1.8 and A_260_/A_230_ < 2.0 were used for subsequent cDNA synthesis. First-strand cDNA was synthesized with TransScript One-Step gDNA Removal and cDNA Synthesis SuperMix (TransGen, Beijing, China) according to the manual, and oligo dT was used for the cDNA synthesis. For each sample, 1 μg of total RNA was used for every 20 μL of the reverse transcription reaction system.

### Real-time quantitative RT-qPCR

RT-qPCR was carried out on an ABI StepOne Real-Time PCR System using TranStart Top Green PCR Super Mix (TransGen). RT-qPCR was carried out on a LightCycler480 System. Reactions were performed in a total volume of 20 μL, which contained 2 μL cDNA template, 0.4 μL each primer, 10 μL 2× Top Green RT-qPCR SuperMix and 6.8 μL ddH2O. The PCR cycling conditions were as follows: 94°C for 30 s and 40 cycles of 95°C for 5 s, 55°C for 15 s and 72°C for 10 s. Melting curve analysis was performed after 40 cycles to test the primer specificity by heating from 65° to 95°C with a stepwise increase of 0.5°C every 10 s. Three technical replicates were used for each sample. Controls without a template were also included. For each gene, the full sample set in each replication was run on the same plate to exclude any technical variation. The amplification efficiencies for all primer pairs were evaluated using fivefold dilutions of the pooled cDNA (1/5, 1/25, 1/125, 1/625, and 1/3125). EASY dilution solution (Takara, Japan) was used for primer dilution.

### Data analysis

The Ct value of each reference gene was used to evaluate its expression level. The amplification efficiencies of the candidate reference genes were calculated according to the following formula: E (%) = (10^−1/slope^ − 1) × 100; the slope was the standard curve of Ct values of the five gradients for each reference gene. The expression stability was evaluated using the ΔCt method (Silver et al., 2006), geNorm (Vandesompele et al., 2002), NormFinder (Andersen et al., 2004), and BestKeeper (Pfaffl et al., 2004), and then comprehensively analyzed using RefFinder (available online: http://omictools.com/reffinders2857.html) (Xie et al., 2012). The ΔCt method was employed to rank the stability of the candidate reference genes by calculating the standard deviation (SD). The gene with the lowest SD was identified as the most stable reference gene (Silver et al., 2006). geNorm makes decisions based on the principle that the expression levels of two appropriate reference genes have a stable ratio across the investigated samples. The expression stability (M) of each candidate gene in geNorm is the variation of the given gene compared with the all other candidate reference genes. The candidate reference genes were ranked based on their M values, and the least stable gene with the highest M value was excluded stepwise. Finally, two genes remained, which were the most stable reference genes. To determine the optimal number of reference genes required for normalization, normalization factors (NFs) were calculated using the geNorm software, with a cut-off value for Vn/n + 1 of 0.15, below which no additional reference gene is required for normalization (Vandesompele et al., 2002). NormFinder ranks candidate reference genes using an ANOVA-based model, which takes inter-group and intra-group relationships into consideration (Andersen et al., 2004). BestKeeper determines the expression stability of candidate reference genes based on SD and CV calculations for Ct values. The most stable reference gene has the lowest SD and CV values (Pfaffl et al., 2004). RefFinder was used to generate a comprehensive ranking based on the geometric mean of the three programs mentioned above (Xie et al., 2012).

### Normalization of gene expression in pistil development and verification of the screened-out reference genes

To validate the selected reference genes, the relative expression levels of three transcription factors, *YAB3, FUL*, and *RPL*, were analyzed during pistil development in radish. The gene sequences were obtained from the genome sequence data of radish (http://www.nodai-genome-d.org/download.html), and primers for the three genes were designed according to the aforementioned methods (primer sequences are listed in Table 1). The specificity of the primers was confirmed by electrophoresis in 2% agarose gels and melting curve analysis. Samples of siliques at different developmental stages were collected as described above. The relative expression level was evaluated using 2^−ΔΔCt^ method. The best reference genes identified by RefFinder (*UP2, GAPDH*, and *UPR*) were used for normalization. The least stable reference gene identified by RefFinder (*GSNOR1*) was also used for normalization. Three biological and three technical replicates were used for the measurements at each sampling point.

**Table 1:** Candidate reference genes and primers used for RT-qPCR in radish, Chinese cabbage, and their distant hybrid.

**Gene**	**Arabidophsis locus description**	**Gene ID**	**Arabidopsis ortholog sequence**	**Forward primer (5′–3′)**	**Reverse primer (5′–3′)**	**Amplicon length**	**Tm (°C)**	**RT-RT-qPCR Efficiency**		
								**Radish**	**Chinese cabbage**	**distant hybrid**
*GSNOR1*	GroES-like zinc-binding dehydrogenase family protein	RSG13314	At5g43940	AAGGTTCGGTCCGCTAC	GAAGGCAGACTTTCTCCAA	181	83.4	0.90	0.94	0.91
*UPR*	Uncharacterized protein family (UPF0041)	RSG14904	At4g22310	GTTCAAGTGGGGAATAAGC	GCAATCTGTTGAGGGTAGG	81	76.8	0.90	0.89	0.91
*DSS1*	deletion of SUV3 suppressor 1(I)	RSG24502	At1g64750	AAGATTGGGTTGAGAAAGAG	GAAGCGAGAAGTCGTCATT	93	80.6	1.03	0.91	0.91
*GAPDH*	Glyceraldehyde-3-phosphate dehydrogenase	RSG33007	At1g13440	GAAATCAAGAAGGCTATCAAGGAG	TTGTCACCAACGAAGTCAGT	101	83.6	0.87	0.87	0.91
*ACTIN2/7*	Actin 2/7	RSG00703	At5g09810	GCATCACACTTTCTACAAC	CCTGGATAGCAACATACAT	155	80	0.93	0.95	0.94
*RPII*	RNA polymerase-II transcription factor	RSG36076	At2g15430	ATCACGCTAAATGGTCTCCT	GCTGCTCTCAATCAAGTCAATC	122	80.5	1.12	0.92	0.90
*TEF_*2*_*	Translation elongation factor 2	RSG09221	At1g56070	AAGAAGATTTGGGCGTTTGG	CCAGCAACAACAGAATCCTT	107	81.5	0.96	0.93	0.90
*TUA*	Tubulin alpha-5	RSG03285	At5g19780	TTCCCTATCCTCGCATCCATTTCA	CCTCGGGTCACACTTAGCCATCA	146	84	0.93	0.94	0.90
*TUB*	Tubulin beta-1	RSG18490	At1g75780	GTCCGGTGCTGGTAATAACTGG	GTGGCATACTTGAAACCCTTGAA	130	84	0.93	0.92	0.92
*UBC9*	Ubiquitin conjugating enzyme 9	RSG00282	At4g27960	GCATCTGCCTCGACATCTTGA	GACAGCAGCACCTTGGAAATG	68	82	1.03	0.90	0.96
*UP2*	Uncharacterized conserved protein UCP022280	RSG21879	At4g26410	AAATTCCTGGGAGGGAAGCTAT	TTCTGTCTCAGGAGCGAAGTCAT	70	80.6	1.00	0.86	0.92
*18SRNA*	18S ribosomal RNA	RSG46827	At3g41768	TACCGTCCTAGTCTCAACCATAA	TTTCAGCCTTGCGACCATAC	130	84.6	0.90	0.92	0.91
*YAB3*	*YABBY* gene family member	RSG26108	At4g00180	GTAGTTTGTTCAAGACCGTAA	GGAGGGAAGAAGAAGAGC	92	81.5	0.92	–	–
*FUL*	MADS-box gene negatively regulated by *APETALA1*	RSG32469	At5g60910	TCAATAGGCAAGTTACTTTCTC	AACCCAATTTTCACTCTGTG	233	82.8	0.94	–	–
*RPL*	Has sequence similarity to the Arabidopsis ovule development regulator Bell1	RSG18312	At5g02030	GGTTTTCTCGGTGGGC	ATCGTAAGTAGGTCTAGGGTG	150	84.3	0.91	–	–

## Results

### Verification of primer efficiency for the candidate reference genes

Based on these selection procedures for the transcriptome sequencing data, 3 genes that had a minor variation in expression were selected (Table S1). Nine traditional reference genes (*GAPDH, RPII, ACTIN2/7, TEF*_*2*_, *18S, TUA, TUB, UBC9*, and *UP2*) from previous studies and three new reference genes (*GSNOR1, UPR*, and *DSS1*) from our analysis were selected as candidate reference genes in our study. Using primers designed for each gene, the PCR amplification specificities of the 12 candidate genes were checked by 2% agarose gel electrophoresis with pistil cDNA samples from radish, Chinese cabbage and their distant hybrid. The results showed that all 12 candidate genes had specific amplification and the product lengths were consistent with the expected lengths in radish, Chinese cabbage and their distant hybrid (Figure S1). Additionally, melting curve analysis of the candidate genes showed a single peak for each gene (Figures S2, S3), which confirmed that the primers for these genes were specific in radish, Chinese cabbage and the distant hybrid. The amplification efficiencies of the 12 candidate genes in different samples varied from 0.86 (*UP2* in Chinese cabbage) to 1.12 (*RPII* in radish) (Table 1, Figures S4, S5).

### Expression stability analysis based on the expression profiles of the candidate reference genes

**Figure 1:**
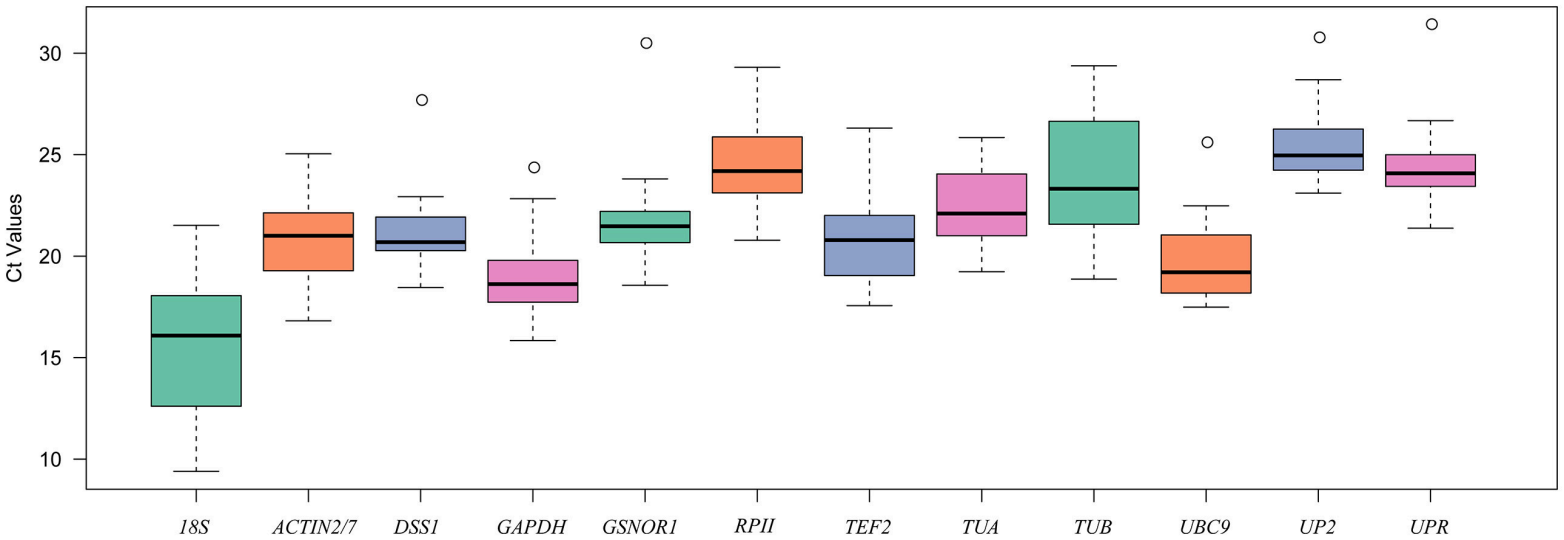
Boxplot analysis of the expression profiles of 12 candidate reference genes across all 28 samples. The line across the box represents the median. The boxes represent the 25/75 percentiles. The whiskers show the maximum and minimum values. The circles indicate outliers.

The expression levels of the 12 candidate reference genes were evaluated in 28 samples collected from different organs in radish, Chinese cabbage and their distant hybrid under abiotic and biotic stress conditions using Ct values. As shown in the boxplot (Figure 1), the Ct values of the 12 candidate reference genes varied from 9.4 (*18S*) to 31.4 (*UPR*). *UP2* had the highest mean Ct value (25.49) with lowest expression abundance among these genes. By contrast, *18S* had the lowest mean value (15.6) with the highest expression abundance. In addition, the expression variation among the 28 samples for each candidate reference gene ranged from 6.61 (*TUA*) to 12.12 (*18S*). These results showed that no candidate reference gene had stable expression under all conditions, and that it is necessary to identify appropriate reference genes for precise normalized expression under specific conditions in *R. sativus*.

The 12 candidate reference genes were then subjected to further analysis based on seven sets of samples: radish organs under normal conditions (RsOs), radish pistils at different developmental stages (RsPs), radish seedlings under biotic (diamondback moth) and abiotic (cold, TuMV) stresses (RsSs), all radish samples (RsAll), Chinese cabbage organs (BrOs), organs of the distant hybrid (HOs) and all samples (All). The ΔCt method, geNorm, NormFinder, BestKeeper, and RefFinder were used to evaluate the expression stability of the candidate reference genes.

In radish organs under normal conditions, *GSNOR1* and *UPR* were the most stable reference genes with M values of 0.34, and *ACTIN2/7* was the next most stable gene with an M value of 0.473 in geNorm analysis. In addition, the recommended number of reference genes for expression normalization was three, with a V3/4 value of 0.119. Therefore, *GSNOR1, UPR*, and *ACTIN2/7* were identified as the optimal reference genes in geNorm analysis. *UPR* was also the most stable reference gene in NormFinder, ΔCt and BestKeeper analysis. The comprehensive ranking order suggested that *UPR, GSNOR1*, and *ACTIN2/7* were the optimal reference genes and *18S* was the least stable reference gene in radish organs (Table 2, Table S2).

**Table 2:** Stability rankings of candidate reference genes in seven sets by ΔCt, BestKeeper, NormFinder, geNorm, and RefFinder.

**Method**	**Ranking order (The 1 st is the most stable, and the 12 th is the least stable)**											
	**1**	**2**	**3**	**4**	**5**	**6**	**7**	**8**	**9**	**10**	**11**	**12**
**Group set**	**RsOs**											
Delta CT	*UPR*	*ACTIN2/7*	*TEF_*2*_*	*GSNOR1*	*UP2*	*DSS1*	*GAPDH*	*UBC9*	*TUB*	*TUA*	*RPII*	*18S*
BestKeeper	*UPR*	*GSNOR1*	*RPII*	*TEF_*2*_*	*GAPDH*	*ACTIN2/7*	*DSS1*	*TUB*	*UP2*	*UBC9*	*TUA*	*18S*
Normfinder	*UPR*	*ACTIN2/7*	*TEF_*2*_*	*UP2*	*GSNOR1*	*DSS1*	*GAPDH*	*UBC9*	*TUB*	*TUA*	*RPII*	*18S*
geNorm	*GSNOR1|UPR*		*ACTIN2/7*	*UP2*	*TEF_*2*_*	*DSS1*	*GAPDH*	*UBC9*	*TUA*	*TUB*	*RPII*	*18S*
Recommended comprehensive ranking	*UPR*	*GSNOR1*	*ACTIN2/7*	*TEF_*2*_*	*UP2*	*DSS1*	*GAPDH*	*RPII*	*UBC9*	*TUB*	*TUA*	*18S*
**Group set**	**RsPs**											
Delta CT	*UP2*	*UPR*	*GAPDH*	*RPII*	*TEF_*2*_*	*UBC9*	*DSS1*	*TUA*	*TUB*	*ACTIN2/7*	*GSNOR1*	*18S*
BestKeeper	*TUA*	*18S*	*ACTIN2/7*	*UP2*	*RPII*	*TUB*	*TEF_*2*_*	*UPR*	*GAPDH*	*DSS1*	*UBC9*	*GSNOR1*
Normfinder	*UP2*	*GAPDH*	*RPII*	*UBC9*	*UPR*	*TEF_*2*_*	*DSS1*	*TUA*	*TUB*	*ACTIN2/7*	*GSNOR1*	*18S*
geNorm	*DSS1|GAPDH*		*UP2*	*UPR*	*UBC9*	*TEF_*2*_*	*RPII*	*TUB*	*TUA*	*ACTIN2/7*	*GSNOR1*	*18S*
Recommended comprehensive ranking	*UP2*	*GAPDH*	*UPR*	*RPII*	*DSS1*	*TUA*	*TEF_*2*_*	*UBC9*	*ACTIN2/7*	*18S*	*TUB*	*GSNOR1*
**Group set**	**RsSs**											
Delta CT	*RPII*	*GAPDH*	*TEF_*2*_*	*UBC9*	*UP2*	*ACTIN2/7*	*DSS1*	*UPR*	*GSNOR1*	*TUA*	*TUB*	*18S*
BestKeeper	*GSNOR1*	*DSS1*	*UPR*	*GAPDH*	*UP2*	*UBC9*	*RPII*	*ACTIN2/7*	*TEF_*2*_*	*TUA*	*TUB*	*18S*
Normfinder	*RPII*	*TEF_*2*_*	*GAPDH*	*UBC9*	*ACTIN2/7*	*UP2*	*DSS1*	*UPR*	*TUA*	*GSNOR1*	*TUB*	*18S*
geNorm	*UP2|UBC9*		*RPII*	*GAPDH*	*DSS1*	*TEF_*2*_*	*ACTIN2/7*	*UPR*	*TUA*	*GSNOR1*	*TUB*	*18S*
Recommended comprehensive ranking	*RPII*	*UBC9*	*GAPDH*	*UP2*	*TEF_*2*_*	*DSS1*	*GSNOR1*	*UPR*	*ACTIN2/7*	*TUA*	*TUB*	*18S*
**Group set**	**RsAll**											
Delta CT	*GAPDH*	*UP2*	*TEF_*2*_*	*DSS1*	*RPII*	*UBC9*	*ACTIN2/7*	*UPR*	*TUA*	*GSNOR1*	*TUB*	*18S*
BestKeeper	*UPR*	*DSS1*	*GAPDH*	*UP2*	*ACTIN2/7*	*RPII*	*GSNOR1*	*TEF_*2*_*	*TUA*	*UBC9*	*TUB*	*18S*
Normfinder	*TEF_*2*_*	*UP2*	*GAPDH*	*RPII*	*ACTIN2/7*	*DSS1*	*UBC9*	*UPR*	*TUA*	*TUB*	*GSNOR1*	*18S*
geNorm	*DSS1|GAPDH*		*UP2*	*UPR*	*UBC9*	*TEF_*2*_*	*RPII*	*ACTIN2/7*	*TUA*	*GSNOR1*	*TUB*	*18S*
Recommended comprehensive ranking	*GAPDH*	*DSS1*	*UP2*	*TEF_*2*_*	*UPR*	*RPII*	*ACTIN2/7*	*UBC9*	*TUA*	*GSNOR1*	*TUB*	*18S*
**Group set**	**BrOs**											
ΔCt	*DSS1*	*UP2*	*GAPDH*	*TEF_*2*_*	*ACTIN2/7*	*RPII*	*UBC9*	*UPR*	*GSNOR1*	*TUB*	*TUA*	*18S*
BestKeeper	*GSNOR1*	*TEF_*2*_*	*DSS1*	*UPR*	*ACTIN2/7*	*UBC9*	*TUA*	*UP2*	*GAPDH*	*RPII*	*TUB*	*18S*
Normfinder	*ACTIN2/7*	*TEF_*2*_*	*UP2*	*DSS1*	*GAPDH*	*RPII*	*UBC9*	*UPR*	*TUB*	*GSNOR1*	*TUA*	*18S*
geNorm	*UP2|GAPDH*		*DSS1*	*TEF_*2*_*	*ACTIN2/7*	*RPII*	*UBC9*	*UPR*	*GSNOR1*	*TUA*	*TUB*	*18S*
Recommended comprehensive ranking	*DSS1*	*UP2*	*TEF_*2*_*	*ACTIN2/7*	*GAPDH*	*GSNOR1*	*UPR*	*UBC9*	*RPII*	*TUA*	*TUB*	*18S*
**Group set**	**HOs**											
ΔCt	*TUA*	*GAPDH*	*UP2*	*RPII*	*TEF_*2*_*	*GSNOR1*	*UBC9*	*ACTIN2/7*	*DSS1*	*UPR*	*TUB*	*18S*
BestKeeper	*DSS1*	*UBC9*	*UPR*	*UP2*	*GAPDH*	*TUA*	*GSNOR1*	*ACTIN2/7*	*RPII*	*TEF_*2*_*	*TUB*	*18S*
Normfinder	*RPII*	*TUA*	*GAPDH*	*TEF_*2*_*	*GSNOR1*	*UP2*	*ACTIN2/7*	*UBC9*	*DSS1*	*UPR*	*TUB*	*18S*
geNorm	*GSNOR1|ACTIN2/7*		*TEF_*2*_*	*RPII*	*GAPDH*	*TUA*	*UP2*	*UBC9*	*DSS1*	*UPR*	*TUB*	*18S*
Recommended comprehensive ranking	*TUA*	*RPII*	*GAPDH*	*GSNOR1*	*ACTIN2/7*	*UP2*	*TEF_*2*_*	*DSS1*	*UBC9*	*UPR*	*TUB*	*18S*
**Group set**	**All**											
ΔCt	*UP2*	*TEF_*2*_*	*GAPDH*	*RPII*	*DSS1*	*UBC9*	*ACTIN2/7*	*UPR*	*TUA*	*GSNOR1*	*TUB*	*18S*
BestKeeper	*UPR*	*DSS1*	*GSNOR1*	*UP2*	*GAPDH*	*UBC9*	*TEF_*2*_*	*RPII*	*TUA*	*ACTIN2/7*	*TUB*	*18S*
Normfinder	*TEF_*2*_*	*UP2*	*GAPDH*	*RPII*	*UBC9*	*DSS1*	*ACTIN2/7*	*TUA*	*UPR*	*GSNOR1*	*TUB*	*18S*
geNorm	*UP2|GAPDH*		*DSS1*	*UBC9*	*TEF_*2*_*	*RPII*	*UPR*	*TUA*	*ACTIN2/7*	*GSNOR1*	*TUB*	*18S*
Recommended comprehensive ranking	*UP2*	*GAPDH*	*TEF_*2*_*	*DSS1*	*UPR*	*UBC9*	*RPII*	*GSNOR1*	*ACTIN2/7*	*TUA*	*TUB*	*18S*

*RsOs, radish organs under normal conditions; RsPs, radish pistils at different developmental stages; RsSs, radish seedlings under biotic (diamondback moth) and abiotic (cold, Tumv) stresses, RsAll: all radish samples; BrOs, of Chinese cabbage organs; Hos, organs of the distant hybrid; All, all samples*.

During radish pistil development, *DSS1* and *GAPDH* were identified as the most stable reference genes with M values of 0.39, and three reference genes were recommended for expression normalization according to the V4/5 value (0.111) by geNorm analysis. *UP2* was the most stable reference gene in NormFinder and ΔCt analysis. In addition, *TUA* was the most stable gene in BestKeeper analysis. The comprehensive ranking indicated that *UP2* and *GAPDH* were the most appropriate reference genes and *GSNOR1* was the least stable reference gene during radish pistil development (Table 2, Table S2).

Under biotic and abiotic stress conditions, *UP2* and *UBC9* were the most stable reference genes with M values of 0.69, and four reference genes were recommended for expression normalization in geNorm analysis. The most stable reference gene was *RPII* according to NormFinder and ΔCt analysis. In addition, *GSNOR1* was the most stable reference gene in BestKeeper analysis. According to the calculations performed by RefFinder, *RPII, UBC9*, and *GAPDH* were the three most stable reference genes in radish samples under biotic and abiotic stresses, whereas *18S* was the least stable reference gene in all of the above evaluation systems (Table 2, Table S2).

Across all of the radish samples, *DSS1* and *GAPDH* were the most stable reference genes, and five reference genes were recommended for expression normalization by geNorm analysis. *GAPDH* was also the best reference gene in ΔCt analysis. However, *TEF*_*2*_ was the best reference gene in NormFinder analysis and *UPR* was recommended by BestKeeper. The comprehensive ranking by RefFinder showed that *GAPDH, DSS1*, and *UP2* were the most stably expressed reference genes across all radish samples, while *18S* was the least stable reference gene (Table 2, Table S2).

In Chinese cabbage organs, *UP2* and *GAPDH* were considered the best reference genes by geNorm analysis, and the appropriate number of genes for normalization was four, with a V4/5 value of 0.126. In contrast, NormFinder recognized *ACTIN2/7* as the most stable reference gene. *DSS1* and *UPR* were the most stable reference genes in ΔCt and BestKeeper analysis, respectively. *DSS1, UP2*, and *TEF*_*2*_ were ranked highly and *18S* was ranked last in the comprehensive analysis by RefFinder (Table 2, Table S2).

In organs of the distant hybrid, *GSNOR1* and *ACTIN2/7* were found to be the best reference genes with M values of 0.26, and four reference genes were recommended for normalization because the pairwise value of V6/7 was 0.142. Conversely, *TUA, DSS1*, and *RPII* were the most stable reference genes in ΔCt, BestKeeper, and NormFinder analysis, respectively. In the comprehensive analysis, *TUA* was determined to be the most stable reference gene in organs of the distant hybrid, and *18S* was identified as the least stable gene (Table 2, Table S2).

**Figure 2:**
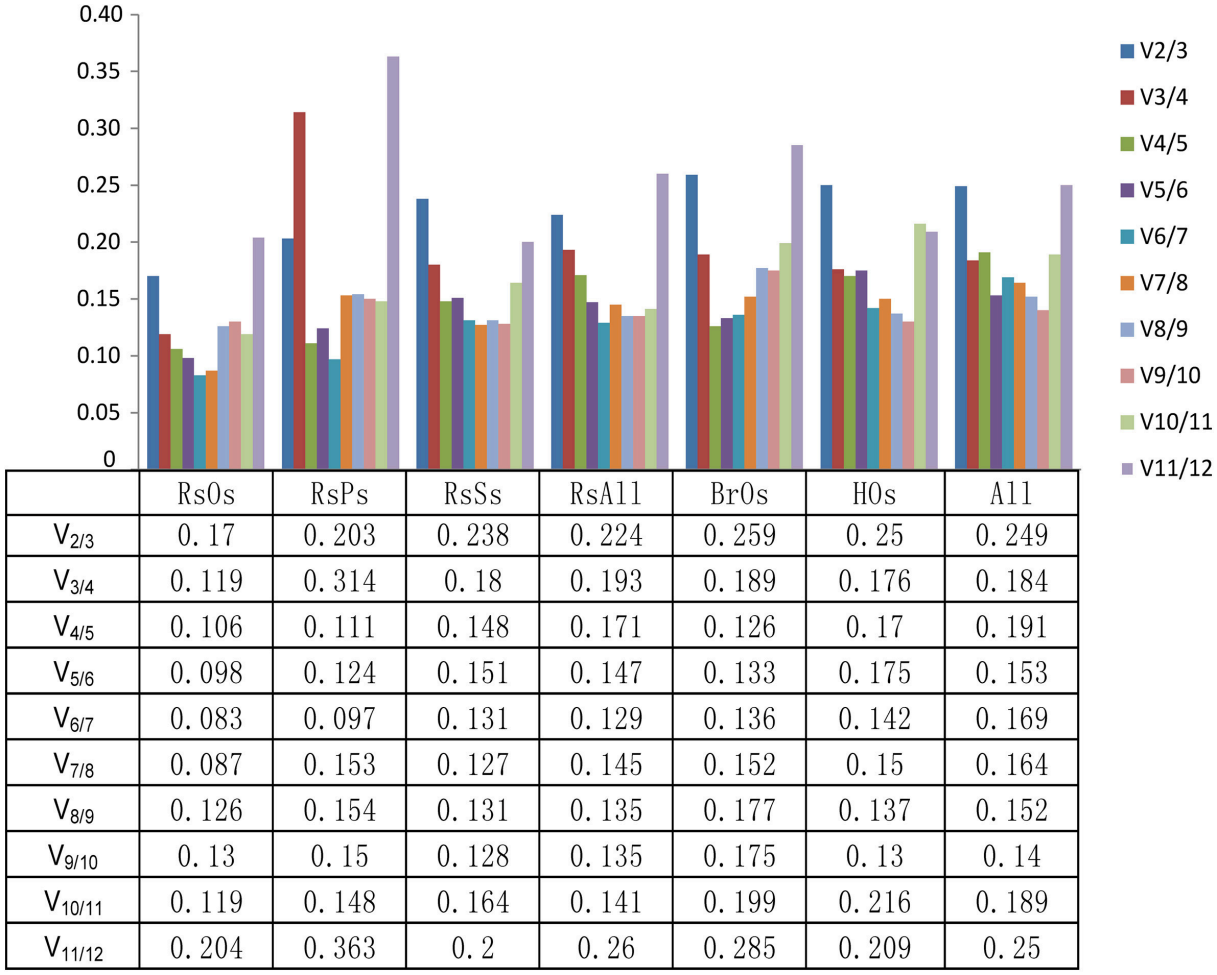
Determination of the optimal number of reference genes. Pairwise variation (Vn/n + 1) was calculated by geNorm to determine the number of reference genes required for accurate normalization in different sample sets. The dashed lines indicate 0.15 was the cut-off value to determine the optimal number of reference genes for gene normalization. RsOs, radish organs under normal conditions; RsPs, radish pistils at different developmental stages; RsSs, radish seedlings under biotic (diamondback moth) and abiotic (cold, TuMV) stresses; RsAll, all radish samples; BrOs, Chinese cabbage organs; HOs, organs of the distant hybrid; All, all samples.

Across all 28 samples, *UP2* and *GAPDH* were identified as the best reference genes with the lowest M values (0.75), whereas *TUB* was identified as the worst reference gene with the highest M value (1.53) in the geNorm analysis (Table 2, Table S2). The results in Figure 2 show that the V8/9 and V9/10 values were 0.152 and 0.14, respectively, which suggests that nine genes are required for reliable normalization when all samples are considered. *GAPDH* was also the best reference gene with the lowest stability value (0.35) according to NormFinder analysis, while *TUB* had the highest stability value (1.43) and was also the least stable reference gene. *UP2* was the most stable reference gene in ΔCt analysis. *UPR* was the most stable reference gene in BestKeeper analysis. In the comprehensive analysis, *UP2, GAPDH*, and *TEF*_*2*_ were the recommended reference genes, and *18S* was the least stable gene in all five ranking lists.

### Validation of candidate reference genes

*YAB3, FUL*, and *RPL*, three important transcription factors during pistil development, were used as examples for expression analysis with *UP2, GAPDH, UPR*, and *GSNOR1* as reference genes for normalization. *UP2, GAPDH*, and *UPR* were the top-ranked genes in RefFinder analysis, and were suggested for accurate expression normalization during pistil development in radish. *GSNOR1* was ranked at the bottom by RefFinder analysis in radish during pistil development (Table 2, Table S2).

**Figure 3:**
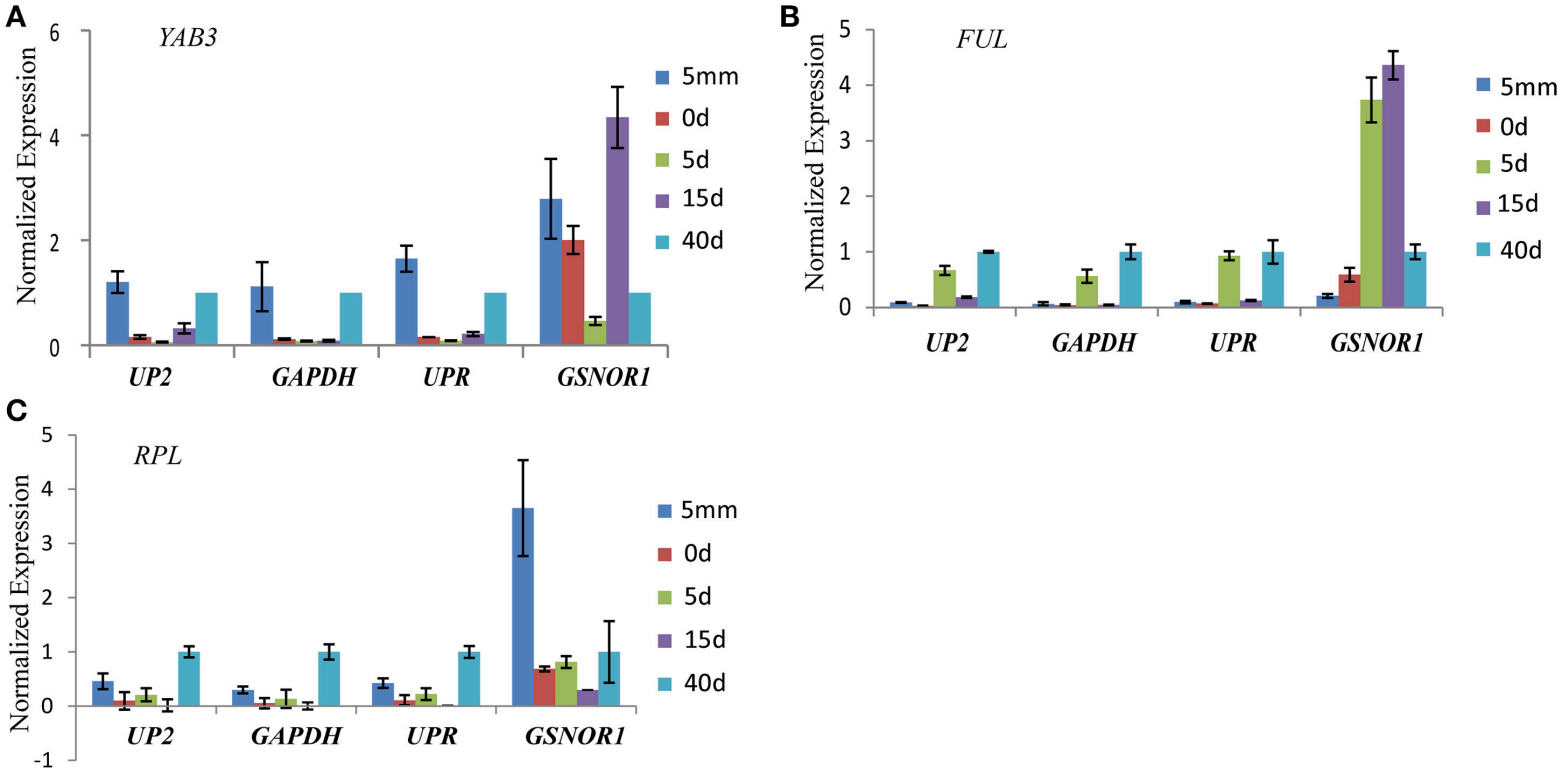
Relative expression levels of *YAB3* **(A)**, *FUL* **(B)**, and *RPL* **(C)** in radish pistil development. The three top-ranked genes (*UP2, GAPDH*, and *UPR*) and the last-ranked genes *GSNOR1* by RefFinder were used for expression normalization. Error bars indicate the standard deviation of three biological replicates.

The expression levels of *YABB3, FUL*, and *RPL* showed similar change patterns when the stable reference genes *UP2, GAPDH*, and *UPR* were used for normalization. In contrast, the expression profiles of *YAB3, FUL*, and *RPL* were distorted when *GSNOR1* was used for normalization. The relative expression levels of *YAB3* and *FUL* at 0 and 15 days were overestimated, and the expression profile of *RPL* at the first stage was abnormally upregulated when *GSNOR1* was used as the reference gene (Figure 3).

## Discussion

RT-qPCR is an essential method for obtaining expression profiles and understanding the biological functions of target genes (Bustin, 2000). The accuracy of RT-qPCR results is mainly dependent on a suitable normalization strategy that selects appropriate reference genes (Vanguilder et al., 2008; Derveaux et al., 2010). Ideally, reference genes should have stable expression in different developmental stages and different tissues or organs, as well as under different experimental conditions, and the expression level should be similar to that of the target gene (Thellin et al., 1999; Suzuki et al., 2000). Reference genes involved in cytoskeleton structure (*ACTIN2/7, TUA, TUB*, and *18S*), protein synthesis (*RPII* and *TEF*_*2*_) and biological metabolic processes (*GAPDH*) are often used as reference genes (Chen et al., 2010; Rebouças et al., 2013). Using genomic and transcriptomic data, novel reference genes such as *UBC9* and *UP2* have also been selected for gene normalization (Schmid et al., 2005; Kwon et al., 2009). In our research, three new genes (*GSNOR, UPR*, and *DSS1*) were selected from radish transcriptome data as candidate reference genes in addition to the seven classical reference genes mentioned above.

However, no candidate reference gene had invariable expression across all samples in our research, which highlights the importance of employing suitable reference genes for particular crops under specific conditions using statistical approaches. In previous studies, the expression stability of reference genes also varied in different species, genotypes, developmental stages, organs, tissues and experimental conditions (Livak and Schmittgen, 2001; Jin et al., 2013). In Chinese cabbage, Qi et al. (2010) found that *EF-1-*α was the best reference gene in a given set of tissues. However, another study showed that *UP1* and *UBC9* were the best choices for vegetative tissues of Chinese cabbage (Chen et al., 2010). In radish, *TEF*_*2*_ and *RPII* performed well in a range of different tissue types (Xu et al., 2012). However, in our research, *UPR, UP2*, and *GAPDH* were ranked first in different organs, pistils at different developmental stages and all radish samples, respectively, while *RPII* was the most suitable reference gene only under biotic and abiotic stresses. Moreover, *18S rRNA*, which is a component of the small subunit of eukaryotic ribosomes (40S), has been considered the most stable gene under various treatment conditions (Jain et al., 2006; Niu X. et al., 2015). However, *18S* was the most unstable reference gene in all samples in our research, which was similar to research by Xu et al. (2012) and in *Pisum sativum* (Die et al., 2010). The *TUB* gene, which participates in cell structural maintenance, was the optimum reference gene under excess salinity and drought conditions in kenaf (Niu X. et al., 2015), but had poor performance in our research, which was similar to results in potato (Reid et al., 2006) and soybean (Hu et al., 2009). Therefore, it is necessary to select appropriate reference genes for normalization according to different species and experimental conditions.

The four computational methods (ΔCt, BestKeeper, NormFinder and geNorm) (Niu L. et al., 2015; He et al., 2016; Li et al., 2016a) for expression stability are based on different algorithms and analytical procedures (Vandesompele et al., 2002; Andersen et al., 2004; Pfaffl et al., 2004; Silver et al., 2006). In our research, we found that the most unstable genes identified by the four algorithms were mostly the same, but the most stable genes were not consistent. This was possibly due to differences among the algorithms. Integrated analysis using different programs can minimize errors in the stability evaluation of candidate reference genes (Li et al., 2016a; Shivhare and Lata, 2016). Here, RefFinder (Xie et al., 2012) was used to combine the results of the four computational methods and generate a comprehensive ranking list for the candidate reference genes. In the comprehensive analysis with RefFinder, the seven traditional reference genes, *GAPDH, ACTIN2/7, UP2, RPII, UBC9, TEF*_*2*_, and *TUA*, and three new reference genes, *GSNOR1, UPR*, and *DSS1*, were found to be the optimal reference genes for gene expression analysis of different species, organs and conditions in the present study.

*GAPDH* is an abundant glycolytic enzyme present in most cell types (Giulietti et al., 2001) and has been extensively used as a reference gene in RT-qPCR experiment. For example, it was recommended for measuring the expression of genes of interest in diverse tissues and genotypes of sugarcane (Iskandar et al., 2004). Furthermore, Mamo indicated that *GAPDH* had consistent changes at different stages of embryonic period (Mamo et al., 2007). Medrano found that *GAPDH* was one of the best genes in combination for embryonic samples (Medrano et al., 2017). Our results were consistent with these previous studies suggesting that *GAPDH* can also be universally used as a reference gene in radish and their relatives, although several reports in animal and plant systems have suggested that *GAPDH* has some limitations as an internal control gene (Sirover, 1999; Nazari et al., 2015). The reference gene *UP2*, encoding an uncharacterized conserved protein, is related to cell structure. Its stability has been verified in many studies (Czechowski et al., 2005; Schmid et al., 2005; Pollier et al., 2014), and in the present research, it also had widely expression stability in all samples. *RPII* was the most stable reference gene under biotic and abiotic stresses and also performed as well as *TUA* in the distant hybrid. In addition, *DSS1* had the least variability across all radish samples and in Chinese cabbage organs. *DSS1* participates in the deletion of SUV3 suppressor 1 and is a 26S proteasome ubiquitin receptor that binds ubiquitin chains (Paraskevopoulos et al., 2014), which may explain why it had stable expression in various conditions. *GSNOR1* is a member of the GroES-like zinc-binding dehydrogenase family and was a suitable reference gene across radish organs. By comparison, *UPR* is an uncharacterized protein and was the best fit for research on radish organs. Accordingly, these reference genes can be selected for gene normalization in different species, tissues and experimental conditions.

Intergenetic and interspecific hybridization are important ways of enriching genetic backgrounds and developing new crop cultivars with novel traits for Cruciferous vegetables (Gueritaine et al., 2002; Darmency et al., 2010; Tonosaki et al., 2013). Radish, Chinese cabbage and their distant hybrid were used as samples for the first time to evaluate the stability of candidate reference genes in this study. Our identification of optimal reference genes will make an important contribution to comparative analyses of target gene expression in these species and their interspecific hybrids.

## Author contributions

Conceived and designed the experiments: XL and MD; Performed the experiments: MD and XZ; Analyzed the data: MD, JW, and YG; Prepared materials: MD, HY, HW, YQ, and JS; Wrote the paper: MD and XL. All of the authors read and approved the final manuscript.

### Conflict of interest statement

The authors declare that the research was conducted in the absence of any commercial or financial relationships that could be construed as a potential conflict of interest.
