# Supplementary material for: Identification of Optimal Reference Genes for Expression Analysis in Radish (Raphanus sativus L.) and Its Relatives Based on Expression Stability
Source: Front Plant Sci. 2017 Sep 15;8:1605. doi: 10.3389/fpls.2017.01605 (PMC5605625; doi:10.3389/fpls.2017.01605)
Supplement: Supplementary file 3 [file Image1.PDF]

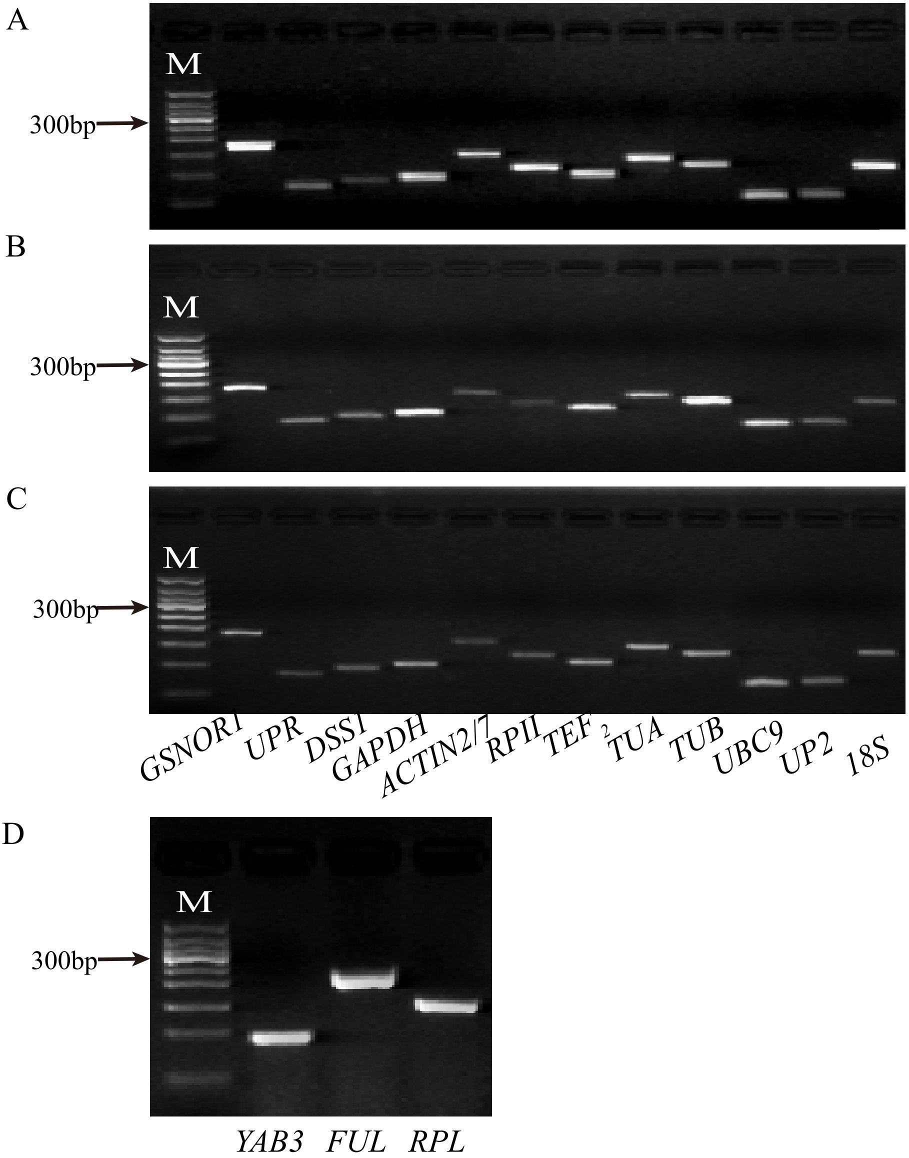

Figure S1 PCR amplification patterns of the 12 candidate reference genes using radish (A), Chinese cabbage (B), the distant hybrid (C) as template respectively, and three verification genes using radish as template (D). ‘M’ represents the marker of 50 bp ladder (from low to up, 50 bp, 100 bp, 150 bp, 200 bp, 250 bp, 300 bp, 350 bp, 400 bp, 450 bp).
