# Supplementary figures and images for: Identification of Optimal Reference Genes for Expression Analysis in Radish (Raphanus sativus L.) and Its Relatives Based on Expression Stability
Source: Front Plant Sci. 2017 Sep 15;8:1605. doi: 10.3389/fpls.2017.01605 (PMC5605625; doi:10.3389/fpls.2017.01605)

A

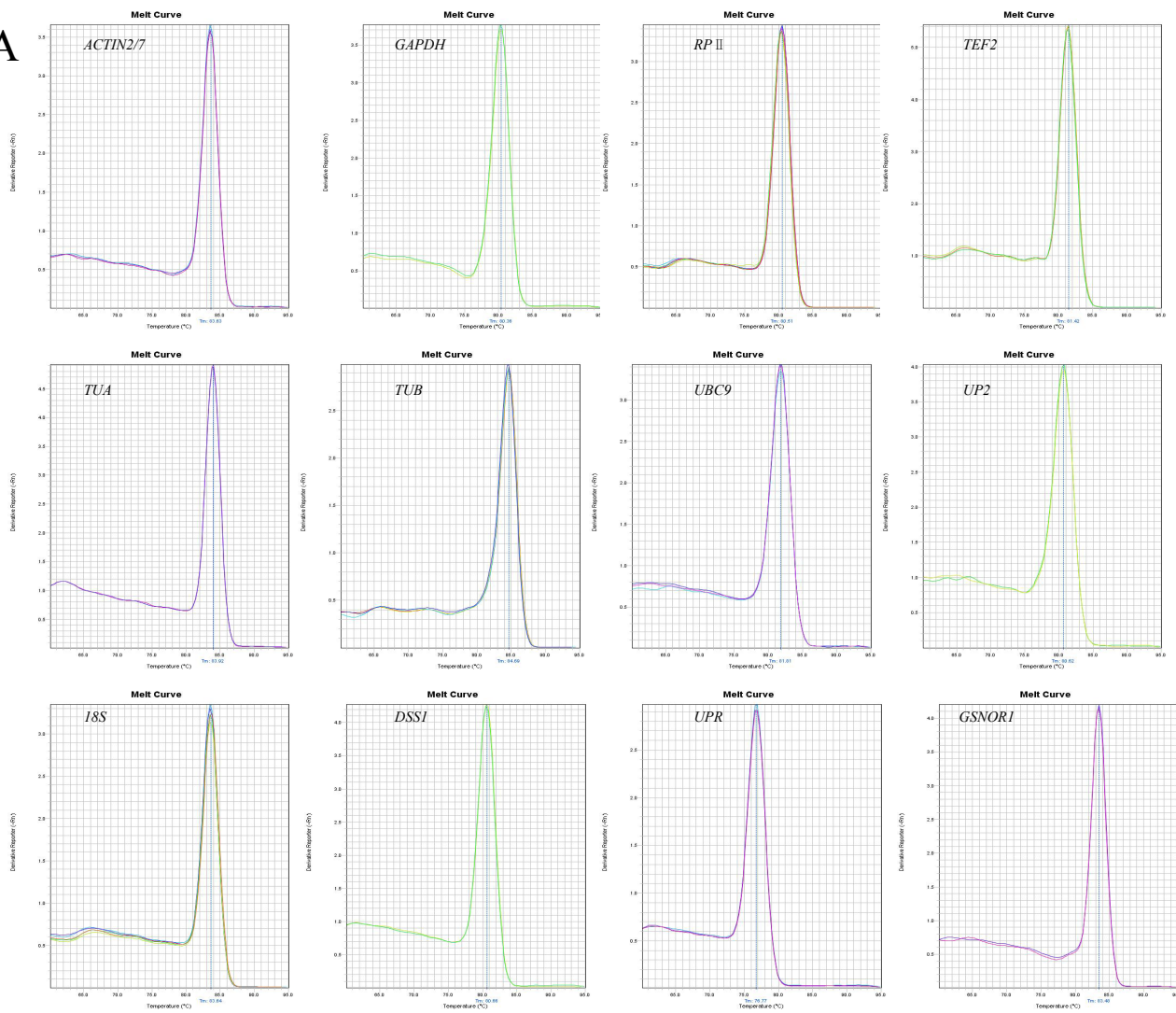

B

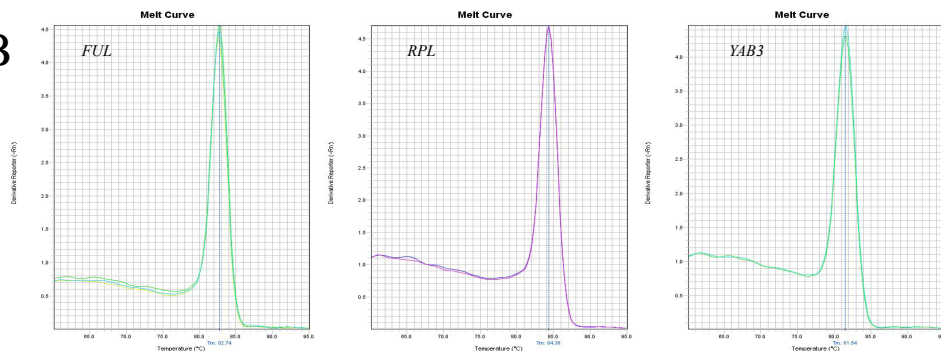

Figure S2 Melting curves of 12 candidate reference genes (A) and 3 verification genes (B) in radish.

Supplement: Supplementary file 4 [file Image2.PDF]

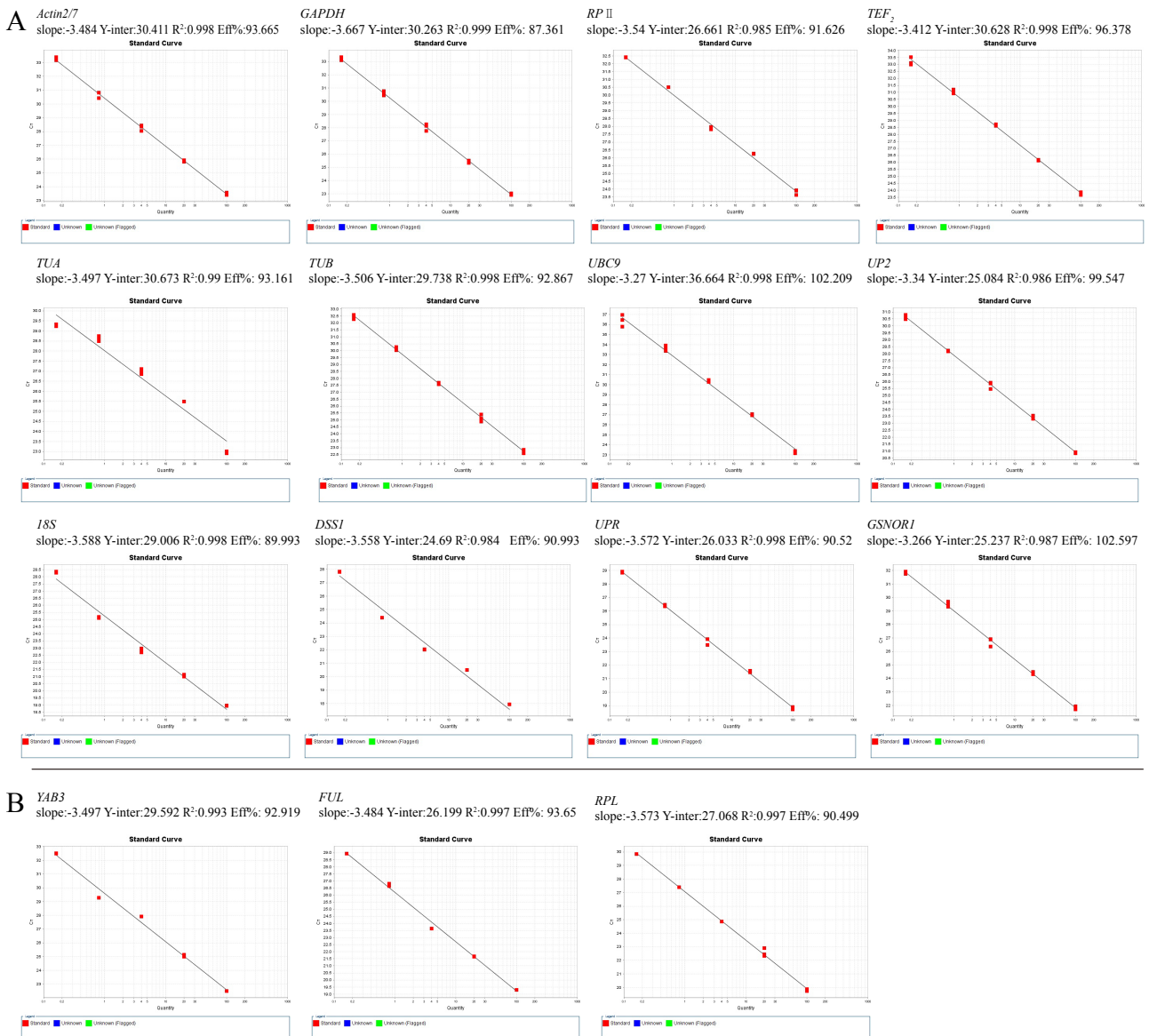

Figure S4 Standard curves of 12 candidate reference genes (A) and 3 verification genes (B) in radish.

Supplement: Supplementary file 6 [file Image4.PDF]
