# Supplementary material for: Identification of Optimal Reference Genes for Expression Analysis in Radish (Raphanus sativus L.) and Its Relatives Based on Expression Stability
Source: Front Plant Sci. 2017 Sep 15;8:1605. doi: 10.3389/fpls.2017.01605 (PMC5605625; doi:10.3389/fpls.2017.01605)
Supplement: Supplementary file 5 [file Image3.PDF]

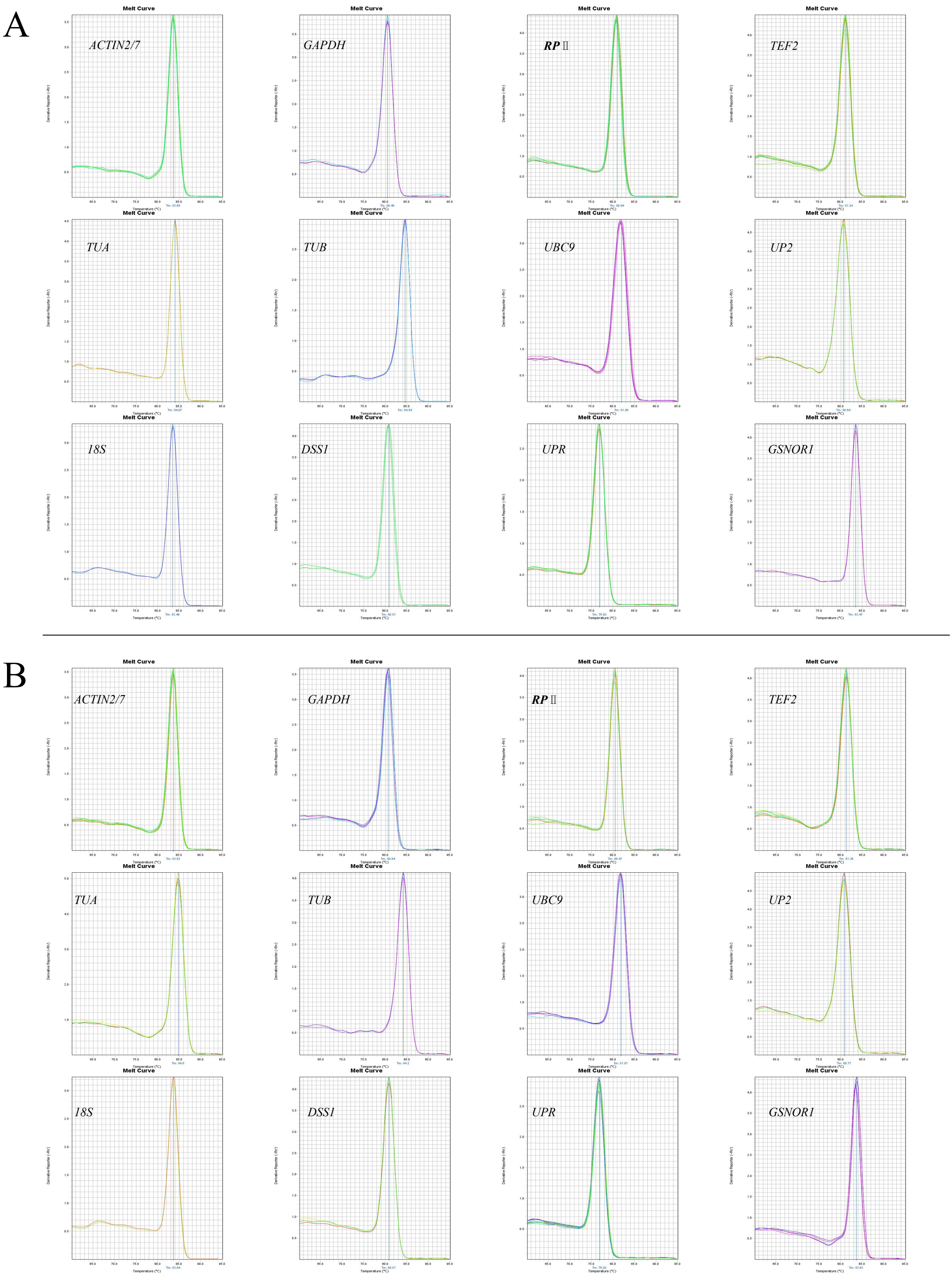

Figure S3 Melting curves of 12 candidate reference genes in Chinese cabbage (A) and the distant hybrid (B).
